# Supplementary material for: The psychological impact of storm Daniel on medical students at the University of Derna in Libya: A cross-sectional study
Source: Glob Ment Health (Camb). 2025 Jul 17;12:e82. doi: 10.1017/gmh.2025.10039 (PMC12322784; doi:10.1017/gmh.2025.10039)
Supplement: Shembesh et al. supplementary material [file S2054425125100393sup001.pdf]

Supplementary materials

This supplementary materials for the following paper:

The psychological impact of storm Daniel on medical students at the University of Derna in Libya: a cross-sectional study

Contents

1. Sociodemographic Questions 2

2. The Generalized Anxiety Disorder 7-item (GAD-7) 3

3. The Generalized Anxiety Disorder 7-item (GAD-7) Arabic translation 4

4. The Patient Health Questionnaire-9 (PHQ-9) 5

5. The Patient Health Questionnaire-9 (PHQ-9) Arabic translation 7

6. Supplementary Table 1. STROBE checklist 9

Socio Demographic Questions

1. State below your Age in numbers.
2. Gender
   - Male
   - Female
3. Choose below the current Year of your medical education
   - Year of study
   - Preparatory yea (ref)
   - First year
   - Second year
   - Third year
   - Fourth year
   - Fifth year
   - Internship
4. Choose below your monthly allowance
   - 0-75 LYD (ref)
   - 76-150 LYD
   - More than 150 LYD
5. What is your living status:
   - With family
   - Alone
6. When Daniel happened, were you displaced from your homes?
   - Yes
   - No

The General Anxiety Disorder-7 (GAD-7)

How often have they been bothered by the following over the past 2 weeks?

1. Feeling nervous, anxious, or on edge
   - Not at all
   - Several days
   - More than half the days
   - Nearly every day
2. Not being able to stop or control worrying
   - Not at all
   - Several days
   - More than half the days
   - Nearly every day
3. Worrying too much about different things
   - Not at all
   - Several days
   - More than half the days
   - Nearly every day
4. Trouble relaxing
   - Not at all
   - Several days
   - More than half the days
   - Nearly every day
5. Being so restless that it's hard to sit still
   - Not at all
   - Several days
   - More than half the days
   - Nearly every day
6. Becoming easily annoyed or irritable
   - Not at all
   - Several days
   - More than half the days
   - Nearly every day
7. Feeling afraid as if something awful might happen
   - Not at all
   - Several days
   - More than half the days
   - Nearly every day

GAD-7 Arabic translation

1. **الشعور بالتوتر، العصبية أو القلق .**
   - ابدا
   - عدة أيام
   - أكثر من نصف األيام
   - كل يوم تقريب

2. **عدم القدرة على ايقاف قلقك وهمومك او السيطرة عليها**
   - ابدا
   - عدة أيام
   - أكثر من نصف األيام
   - كل يوم تقريب

3. **القلق و الهم الزائد حول عدة أمور**
   - ابدا
   - عدة أيام
   - أكثر من نصف األيام
   - كل يوم تقريب

4. **صعوبة في االسترخاء .**
   - ابدا
   - عدة أيام
   - أكثر من نصف األيام
   - كل يوم تقريب

5. **الشعور بعدم االستقرار لدرجة تصعب عليك فيها الجلوس بال حركة**
   - ابدا
   - عدة أيام
   - أكثر من نصف األيام
   - كل يوم تقريب

6. **االنفعال أو االنزعاج بسهولة**
   - ابدا
   - عدة أيام
   - أكثر من نصف األيام
   - كل يوم تقريب

7. **الشعور بالخوف وكأن شيء مريع قد يحدث لك.**
   - ابدا
   - عدة أيام
   - أكثر من نصف األيام
   - كل يوم تقريب

The Patient Health Questionnaire-9 (PHQ-9)

Over the last 2 weeks, how often have you been bothered by any of the following problems? (Use "✓" to indicate your answer)

1. little interest or pleasure in doing things?
   - Not at all
   - Several days
   - More than half the days
   - Nearly every day
2. Feeling down, depressed, or hopeless?
   - Not at all
   - Several days
   - More than half the days
   - Nearly every day
3. Trouble falling or staying asleep, or sleeping too much?
   - Not at all
   - Several days
   - More than half the days
   - Nearly every day
4. Feeling tired or having little energy?
   - Not at all
   - Several days
   - More than half the days
   - Nearly every day
5. Poor appetite or overeating?
   - Not at all
   - Several days
   - More than half the days
   - Nearly every day

6. Feeling bad about yourself — or that you are a failure or have let yourself or your family down?
   - Not at all
   - Several days
   - More than half the days
   - Nearly every day
7. Trouble concentrating on things, such as reading the newspaper or watching television?
   - Not at all
   - Several days
   - More than half the days
   - Nearly every day
8. Moving or speaking so slowly that other people could have noticed? Or so fidgety or restless that you have been moving a lot more than usual?
   - Not at all

- Several days
- More than half the days
- Nearly every day

9. Thoughts that you would be better off dead, or thoughts of hurting yourself in some way?
   - Not at all
   - Several days
   - More than half the days
   - Nearly every day

PHQ-9 Arabic translation

1. **قلة االهتمام أو المتعة عند القيام باألشياء**
- ابدا
- عدة أيام
- أكثر من نصف األيام
- كل يوم تقريب

2. **الشعور بالضيقة أو االكتئاب أو اليأس**
- ابدا
- عدة أيام
- أكثر من نصف األيام
- كل يوم تقريب

3. **صعوبات في النوم أو في االستمرار في النوم أو كثرة النوم**
- ابدا
- عدة أيام
- أكثر من نصف األيام
- كل يوم تقريب

4. **الشعور بالتعب أو قلة النشاط**
- ابدا
- عدة أيام
- أكثر من نصف األيام
- كل يوم تقريب

5. **قلة الشهية أو شراهة األكل**
- ابدا
- عدة أيام
- أكثر من نصف األيام
- كل يوم تقريب

6. **الشعور بعدم الرضا عن نفسك أو الشعور بأنك إنسان فاشل أو بأنك خذلت نفسك أو عائلتك**
- ابدا
- عدة أيام
- أكثر من نصف األيام
- كل يوم تقريب

7. **صعوبات في التركيز على األشياء كقراءة الجريدة أو مشاهدة التلفاز**
- ابدا
- عدة أيام
- أكثر من نصف األيام
- كل يوم تقريب

8. **التحرك أو التحدث ببطء شديد لدرجة ملحوظة، أو العكس التململ وعدم القدرة على االستقرار لدرجة التحرك من مكان آلخر أكثر من المعتاد**
- ابدا
- عدة أيام
- أكثر من نصف األيام
- كل يوم تقريب

9. التفكير بأنه من األفضل لك الموت أو التفكير بإيذاء نفسك بطريقة ما.

- ابدا
- عدة أيام
- أكثر من نصف األيام
- كل يوم تقريب

Supplementary Table 1. STROBE Statement

	Item No	Recommendation	Page No
**Title and abstract**	1	(*a*) Indicate the study's design with a commonly used term in the title or the abstract	1
		(*b*) Provide in the abstract an informative and balanced summary of what was done and what was found	1
**Introduction**			
Background/rationale	2	Explain the scientific background and rationale for the investigation being reported	2
Objectives	3	State specific objectives, including any prespecified hypotheses	2
**Methods**			
Study design	4	Present key elements of study design early in the paper	3
Setting	5	Describe the setting, locations, and relevant dates, including periods of recruitment, exposure, follow-up, and data collection	3
Participants	6	(*a*) *Cohort study*—Give the eligibility criteria, and the sources and methods of selection of participants. Describe methods of follow-up<br><br>*Case-control study*—Give the eligibility criteria, and the sources and methods of case ascertainment and control selection. Give the rationale for the choice of cases and controls<br><br>*Cross-sectional study*—Give the eligibility criteria, and the sources and methods of selection of participants	3
		(*b*) *Cohort study*—For matched studies, give matching criteria and number of exposed and unexposed<br><br>*Case-control study*—For matched studies, give matching criteria and the number of controls per case	-
Variables	7	Clearly define all outcomes, exposures, predictors, potential confounders, and effect modifiers. Give diagnostic criteria, if applicable	3-4

Data sources/ measurement	8*	For each variable of interest, give sources of data and details of methods of assessment (measurement). Describe comparability of assessment methods if there is more than one group	*3-4*
Bias	9	Describe any efforts to address potential sources of bias	9
Study size	10	Explain how the study size was arrived at	3
Quantitative variables	11	Explain how quantitative variables were handled in the analyses. If applicable, describe which groupings were chosen and why	4
Statistical methods	12	(*a*) Describe all statistical methods, including those used to control for confounding	4
		(*b*) Describe any methods used to examine subgroups and interactions	4
		(*c*) Explain how missing data were addressed	-
		(*d*) *Cohort study*—If applicable, explain how loss to follow-up was addressed<br>*Case-control study*—If applicable, explain how matching of cases and controls was addressed<br>*Cross-sectional study*—If applicable, describe analytical methods taking account of sampling strategy	3
		(*e*) Describe any sensitivity analyses	-

Results

Participants	13*	(a) Report numbers of individuals at each stage of study—eg numbers potentially eligible, examined for eligibility, confirmed eligible, included in the study, completing follow-up, and analysed	4
		(b) Give reasons for non-participation at each stage	-
		(c) Consider use of a flow diagram	-
Descriptive data	14*	(a) Give characteristics of study participants (eg demographic, clinical, social) and information on exposures and potential confounders	4 Table 1
		(b) Indicate number of participants with missing data for each variable of interest	-
		(c) *Cohort study*—Summarise follow-up time (eg, average and total amount)	-

Outcome data	15*	*Cohort study*—Report numbers of outcome events or summary measures over time	-
		Case-control study—Report numbers in each exposure category, or summary measures of exposure	-
		Cross-sectional study—Report numbers of outcome events or summary measures	*4-5*
Main results	16	(*a*) Give unadjusted estimates and, if applicable, confounder-adjusted estimates and their precision (eg, 95% confidence interval). Make clear which confounders were adjusted for and why they were included	-
		(*b*) Report category boundaries when continuous variables were categorized	3-4
		(*c*) If relevant, consider translating estimates of relative risk into absolute risk for a meaningful time period	-
Other analyses	17	Report other analyses done—eg analyses of subgroups and interactions, and sensitivity analyses	4
**Discussion**			
Key results	18	Summarise key results with reference to study objectives	6-8
Limitations	19	Discuss limitations of the study, taking into account sources of potential bias or imprecision. Discuss both direction and magnitude of any potential bias	9
Interpretation	20	Give a cautious overall interpretation of results considering objectives, limitations, multiplicity of analyses, results from similar studies, and other relevant evidence	6-8
Generalisability	21	Discuss the generalisability (external validity) of the study results	9
**Other information**			
Funding	22	Give the source of funding and the role of the funders for the present study and, if applicable, for the original study on which the present article is based	9
